# Paroxysmal Sympathetic Hyperactivity Complicated by Rhabdomyolysis and Acute Kidney Injury Following Spontaneous Pontine Hemorrhage

**DOI:** 10.7759/cureus.88602

**Published:** 2025-07-23

**Authors:** Sophie Jia Qian Koh, Yu Tung Lo, Vincent Diong Weng Nga

**Affiliations:** 1 Neurosurgery, National University Hospital, Singapore, SGP

**Keywords:** autonomic nervous system, brain injuries, critical care, dysautonomia, intracerebral hemorrhage, paroxysmal sympathetic hyperactivity

## Abstract

Paroxysmal sympathetic hyperactivity (PSH) is a potentially life-threatening clinical syndrome characterized by sudden, episodic increases in sympathetic nervous system activity. While commonly described after traumatic brain injury, it is a rare and under-recognized complication of intracerebral hemorrhage (ICH). Diagnosis is challenging in the acute setting due to its nonspecific presentation and constellation of symptoms. The pathophysiology remains poorly understood, and no standardized diagnosis or treatment guidelines exist.

We describe a case of a 50-year-old male patient with spontaneous pontine ICH who developed PSH within a week of admission, with hyperthermia, hypertension, tachycardia, and dystonic posturing. Despite multimodal therapy with bromocriptine, baclofen and clobazam, the patient developed complications of rhabdomyolysis likely from drug-refractory paroxysmal rigidity and acute kidney injury requiring dialysis.

This case highlights the diagnostic and therapeutic challenges of PSH in neurocritical care, underscoring the necessity of heightened clinical suspicion, exclusion of mimics, and early individualized treatment to optimise outcomes.

## Introduction

Paroxysmal sympathetic hyperactivity (PSH) is a clinical syndrome characterized by sudden and episodic sympathetic overactivity, typically occurring after severe brain injury. First described by Penfield in 1929 [1] as "diencephalic autonomic epilepsy", the syndrome manifests as recurrent episodes of excessive autonomic activation, including hyperthermia, hypertension, tachycardia, tachypnea, diaphoresis, and dystonic posturing [2]. While most frequently reported after traumatic brain injury (TBI), PSH has been recognized across diverse neurological insults, including stroke, anoxic brain injury, and intracranial hemorrhage [3].

Despite its clinical significance, PSH remains underdiagnosed due to its variable presentation and overlap with conditions such as sepsis, seizures, and malignant hyperthermia [4]. The lack of standardized diagnostic criteria further complicates early identification. Although uncommon, PSH is clinically significant because uncontrolled sympathetic storms can increase metabolic demand, precipitate complications such as rhabdomyolysis or cardiac strain, prolong intensive care stays, and worsen overall morbidity. Timely control of PSH is therefore important, as delayed treatment may result in secondary organ injury and poorer outcomes. This diagnostic uncertainty is reflected in the proliferation of terminology, with Perkes et al. [2] identifying 349 cases described under 31 different terms, including "dysautonomia" and "sympathetic storm". They proposed the unifying term PSH, which has since been widely adopted in contemporary literature. The pathophysiology remains incompletely understood, with theories implicating disrupted cortical inhibition or exaggerated spinal sympathetic reflexes [5].

To aid diagnosis, the PSH Assessment Measure (PSH-AM) score was developed, comprising a Clinical Feature Scale and a Diagnosis Likelihood Tool. This tool incorporates clinical features to evaluate symptom frequency, severity, and treatment response, and has shown moderate inter-rater reliability and strong discriminant validity in differentiating PSH from mimics [3].

Current management remains empirical, typically combining gamma-aminobutyric acid (GABA)ergic agents with sympathetic modulators, though evidence-based protocols are lacking [6]. A scoping review found monotherapy generally ineffective, with inconsistent treatment efficacy across pharmacologic classes [7]. Conventional oral therapies, including sedatives, muscle relaxants, antiadrenergic agents, and baclofen, often fail to adequately control PSH [2]. Case series highlight variable benefit from baclofen and benzodiazepines, with minimal impact on attack frequency or intensity [8]. The condition's variable presentation across different brain injury types creates particular therapeutic challenges, with limited guidance specific to hemorrhagic stroke compared to traumatic etiologies.

This case of PSH following spontaneous pontine hemorrhage contributes to the growing literature by highlighting: (i) unique diagnostic considerations in brainstem hemorrhage, where direct autonomic center involvement may drive severe presentations; and (ii) therapeutic dilemmas in ICH-associated PSH management.

## Case presentation

**Figure 1:**
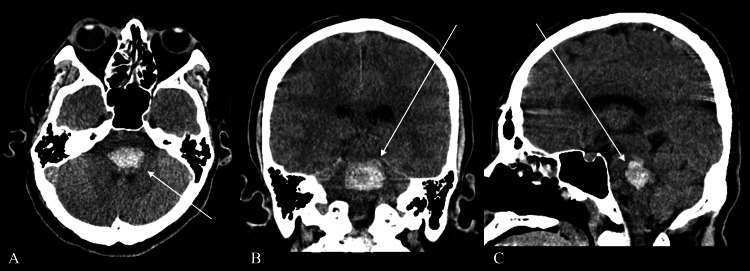
CT brain images CT: Computed Tomography (A) Axial, (B) Coronal, and (C) Sagittal views, demonstrating a hyperdense acute intracerebral hematoma centered in the pons with mass effect but no upstream hydrocephalus

A man in his 50s with a history of poorly-controlled hypertension, secondary to medication noncompliance, presented to the Emergency Department with a sudden-onset headache, nausea, and generalized tonic-clonic seizures. On examination, he was drowsy, with a Glasgow Coma Scale (GCS) of three, and had a markedly elevated systolic blood pressure of >250 mmHg. Neuroimaging with Computed Tomography (CT) of the brain revealed an acute pontine hematoma (Figure 1).

He was admitted to the intensive care unit for ventilatory support, blood pressure control, and neuroprotective measures. An interval CT at 24 hours showed a stable hematoma with no hydrocephalus. Sedation was weaned, and his neurological status improved to GCS E4VTM6. However, the patient exhibited quadriplegia with no voluntary movement of the upper or lower limbs. He was in a locked‑in state and able to communicate solely through vertical eye movements and blinking in response to commands. In view of the persistent bulbar dysfunction with absent protective airway reflexes and a high risk of aspiration attributable to the pontine lesion, he was deemed unsuitable for extubation.

**Figure 2:**
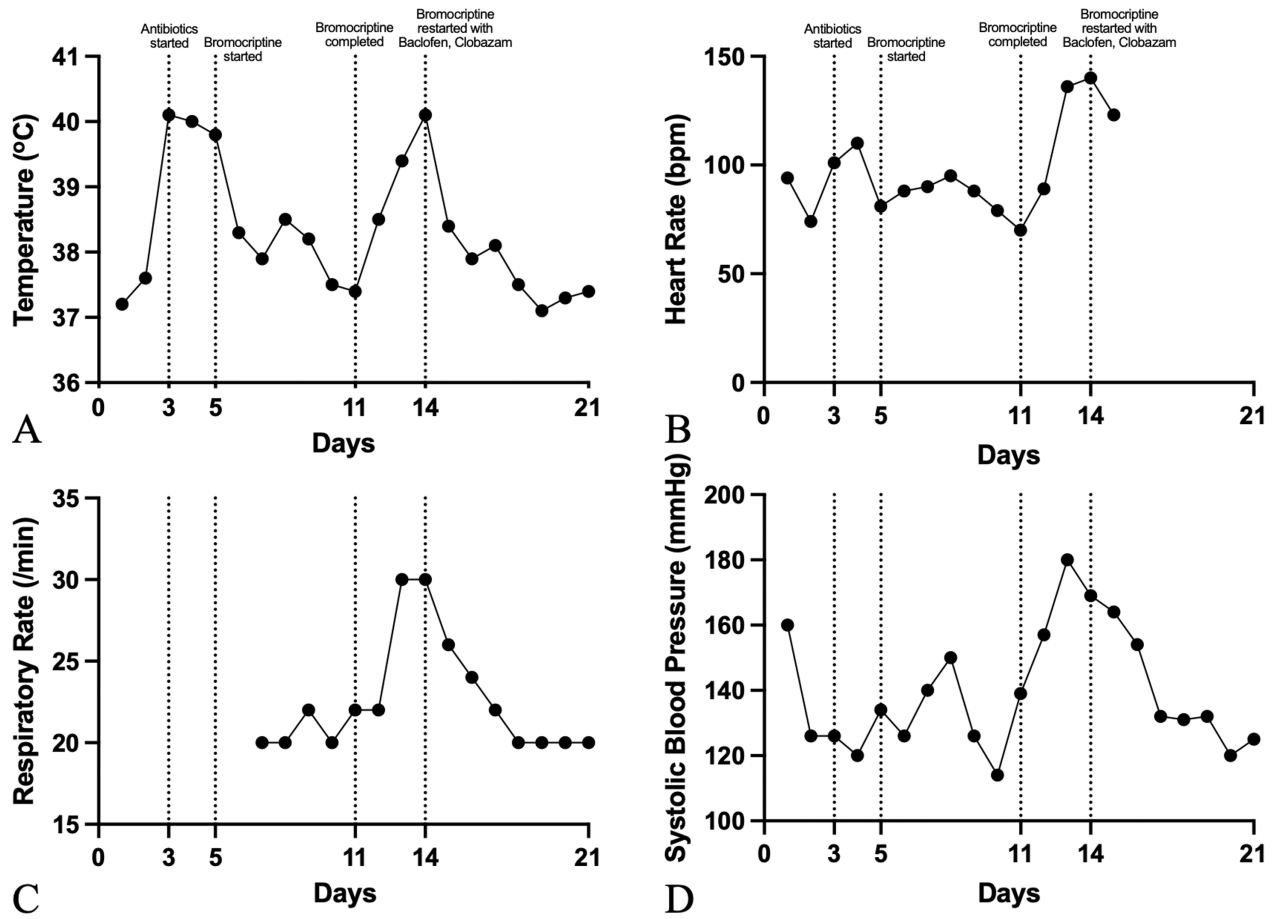
Clinical trends in the patient's (A) temperature, (B) heart rate, (C) respiratory rate, and (D) systolic blood pressure over a 21-day period Antibiotics were initiated on day three for suspected chest infection. Bromocriptine was started on day three at 2.5 mg orally every 12 hours, which was titrated to 5 mg every eight hours over 48 hours, based on clinical response and tolerability. Bromocriptine was selected as the initial dopaminergic agent given its established use in published PSH case reports, favorable safety profile in critically-ill patients, and reliable oral administration route. Compared with other dopaminergic agents such as amantadine or levodopa, bromocriptine has more consistent documentation in attenuating hyperthermic autonomic storms with a lower risk of agitation or hypotension in the neurocritical care setting. Following initiation, there was a gradual improvement in fever and autonomic parameters. On day 11, bromocriptine was completed with resolution of fever. However, on day 13, there was a recurrence of hyperthermia accompanied by autonomic signs, including tachycardia, tachypnea, and hypertension. On day 14, bromocriptine was restarted in combination with baclofen and clobazam. These agents had initially been withheld because the patient demonstrated a partial response to bromocriptine monotherapy, and early polypharmacy was avoided to reduce sedative burden and preserve the ability to perform serial neurological assessments. With recurrent paroxysms despite adequate bromocriptine dosing, baclofen (initiated at 5 mg orally every eight hours and uptitrated to 10 mg every eight hours) and clobazam (initiated at 5 mg orally at night and titrated to 10 mg twice daily) were introduced on day 14, providing additional GABAergic modulation and spasticity control. Following this stepwise escalation, there was gradual stabilization of all parameters. Note: Respiratory rate was not recorded until day seven as the patient was on controlled mechanical ventilation prior to this.

Subsequently, he developed hyperthermia on day three (up to 40.1 °C) and mildly elevated inflammatory markers, with total white blood cell (TWBC) of 12.94 x10^9^/L, C-reactive protein (CRP) levels at 61.3 mg/L, and procalcitonin of 2.7 µg/L. Broad-spectrum antibiotics were initiated for a presumed chest infection. Despite two days of targeted antibiotic therapy for Klebsiella pneumoniae on respiratory cultures, the patient continued to experience fever spikes (up to 40 °C) and was empirically started on bromocriptine, a dopamine agonist to suppress sympathetic overdrive for central fever. As his temperature and inflammatory markers normalized (TWBC 10.3 x10^9^/L, CRP 47.2 mg/L, and procalcitonin 0.2 µg/L), bromocriptine was tapered. However, hyperthermia recurred upon cessation, accompanied by fluctuating consciousness, extensor posturing, and intermittent episodes of tachycardia (up to 140 bpm), tachypnea (up to 30/min) and systolic hypertension (up to 180mmHg), even in the absence of stimuli (Figure 2).

Bromocriptine was selected as the first‑line dopaminergic agent in view of its established use in published PSH case reports, its favorable safety profile in critically ill patients, and the availability of an oral formulation that allowed reliable administration via nasogastric tube. Compared to other dopaminergic agents such as amantadine or levodopa, bromocriptine has more consistent documentation in the management of hyperthermic autonomic storms and less potential for exacerbating agitation or precipitating hypotension in a neurocritical care setting.

There were no clinical or EEG evidence of seizure activity even during episodes of intermittent posturing. Further investigations excluded sepsis and metabolic derangements. Given the temporal association and stereotypical pattern of autonomic features, PSH was suspected. Opioid therapy was not trialed in this case because of concerns regarding respiratory depression and the need to preserve reliable neurological examinations in a patient with brainstem involvement. Instead, bromocriptine was reinitiated with the addition of baclofen to address dystonia and autonomic instability, with slight symptom improvement.

Despite these, the patient developed rhabdomyolysis and oliguric acute kidney injury, evidenced by rising serum creatinine (545 µmol/L), urea (43.6 mmol/L), and creatine kinase (23,000 U/L) levels. Myoglobinuria was confirmed on urinalysis. Clobazam was introduced, and supportive measures, including hydration and renal replacement therapy, were initiated. This led to biochemical and clinical improvement.

By day 21, the autonomic episodes resolved with multimodal therapy. However, neurological recovery remained poor with fluctuating consciousness levels and the patient was unable to consistently achieve a GCS motor score beyond M4 (localizing to pain). Persistent hypercapnia and recurrent aspiration pneumonias necessitated tracheostomy creation. Despite aggressive pulmonary hygiene, he developed septic shock from hospital-acquired multidrug-resistant pneumonia. After family discussions, comfort-focused care was initiated and the patient passed on two months post-admission from hospital-acquired pneumonia.

Informed consent for publication was obtained from the patient’s legal next‑of‑kin.

## Discussion

PSH following spontaneous intracerebral hemorrhage represents a diagnostically complex and clinically significant phenomenon, which is underrecognized in neurocritical care. While well-documented in TBI, its occurrence after brainstem hemorrhage is rarely reported and poses unique management challenges. We present a case of PSH secondary to pontine hemorrhage, complicated by rhabdomyolysis and acute kidney injury, to highlight the diagnostic pitfalls and therapeutic strategies.

Although this patient’s death was not directly attributable to PSH but to poor neurological recovery from the primary pontine injury and hospital‑acquired infections, the syndrome may have contributed indirectly to the overall outcome. PSH can prolong intensive care requirements by delaying ventilator weaning, increasing sedative needs, and precipitating secondary complications such as rhabdomyolysis, which in turn may lengthen hospital stay and increase the risk of nosocomial infections. While this association is clinically plausible, it remains speculative, as no direct causal link between PSH and infection risk has been established.

Pathophysiology of PSH and anatomical correlates

The pathophysiology of PSH remains incompletely understood, with several competing theories proposed. Early epileptogenic-based hypotheses have been discounted, after studies failed to demonstrate seizure activity correlating with autonomic storms [9]. The prevailing disconnection theory posits that brain injuries disrupt inhibitory cortical control (including the insula, cingulate cortex, middle temporal cortex, and dorsolateral prefrontal cortex) over subcortical autonomic centers (hypothalamus, diencephalon, and brainstem) [2], leading to unregulated sympathetic outflow. However, this model alone fails to fully account for the episodic, stimulus-sensitive nature of PSH [10].

To address this, the Excitatory:Inhibitory Ratio (EIR) model proposes a two-stage process [11] where the loss of descending inhibition permits the formation of hyperexcitable spinal circuits, and afferent dysregulation transforms non-noxious stimuli into perceived threats, triggering exaggerated sympathetic and motor responses.

**Figure 3:**
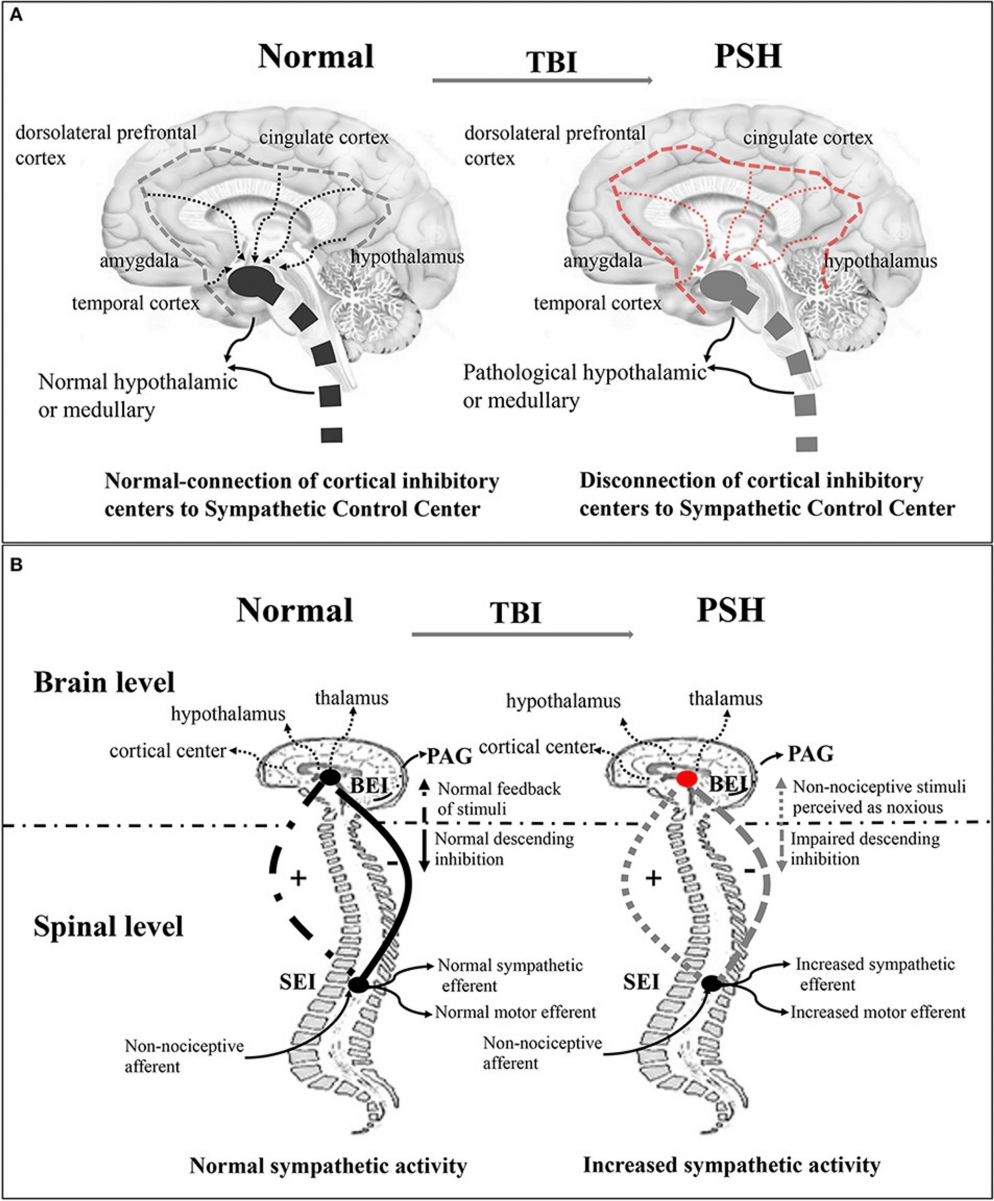
Pathogenesis of PSH PSH: paroxysmal sympathetic hyperactivity; EIR model: Excitatory Inhibitory Ratio model; TBI: traumatic brain injury (A) In a healthy brain, cortical inhibitory centers (e.g., insula and cingulate cortex) regulate hypothalamic and brainstem sympathetic centers. In TBI, this cortical input is disrupted, resulting in unchecked autonomic output. (B) At the spinal level, the normal balance between descending inhibition and ascending sensory feedback maintains stable sympathetic tone. In PSH, this balance is lost, creating a feedback loop in which benign stimuli provoke outsized sympathetic discharge. Figure from Zheng et al. [12].

A visual summary by Zheng et al. [12] shows both the disconnection and EIR model (Figure 3). 

Our patient’s pontine hemorrhage likely disrupted key autonomic nuclei including the parabrachial complex, locus coeruleus, and ventrolateral medulla - integral to sympathetic and cardiovascular regulation [13] - leading to unchecked sympathetic hyperactivity. This disruption underpins the severe autonomic instability observed. The patient’s response to GABAergic agents (baclofen, clobazam) further supports the role of impaired inhibitory pathways.

Clinical observations suggest that specific injury patterns correlate with PSH development. Lesions in the periventricular white matter, corpus callosum, diencephalon, brainstem (as in our case) [4] are at higher risk than those with purely cortical or subcortical injuries. However, challenges remain in precisely defining the contributions of specific gray matter structures, due to the diffuse nature of most brain injuries [14] and lack of standardized neuroimaging protocols [5].

Diagnostic challenges

The diagnosis of PSH presents a significant clinical challenge in neurocritical care settings. While physiological sympathetic activation occurs normally after acute brain injury [15], PSH represents a pathological extreme that can lead to secondary neurological injury and systemic complications, including cardiac strain and death [3].

Diagnosing PSH is challenging as its core clinical features, such as tachycardia, hypertension, tachypnea, hyperthermia, and dystonia, are nonspecific and overlap substantially with common ICU conditions such as sepsis, seizures, and drug reactions (i.e. neuroleptic malignant syndrome) [4]. The absence of definitive biomarkers further complicates diagnosis, which remains primarily clinical and requires exclusion of mimics which may lead to a diagnostic delay [5]. Although autonomic testing modalities such as heart rate variability analysis, sympathetic skin response, or baroreflex sensitivity could theoretically provide supportive evidence of sympathetic overactivity, these were not pursued in our patient because they are not routinely available in the acute ICU setting, require patient cooperation and stable conditions to obtain interpretable results, and have limited validated diagnostic utility for PSH in critically-ill patients.

To objectify the diagnosis, the PSH-AM [3] was applied in this case. Using the Clinical Feature Scale and Diagnosis Likelihood Tool, our patient scored 15 points, placing him in the probable PSH category (scores ≥17 indicate probable PSH; 8-16 possible; ≤7 unlikely). The PSH‑AM has been reported to have a sensitivity of approximately 0.94 and specificity of 0.80 for PSH when applied in traumatic brain injury cohorts, and although data in hemorrhagic stroke are limited, these values support its utility in differentiating PSH from mimics. In our case, calculating the PSH‑AM score helped formalize diagnostic reasoning, track symptom clusters objectively, and provided a structured framework to justify targeted therapy. The PSH-AM tool provides a framework for standardizing diagnosis, though its clinical implementation remains limited. Our experience emphasizes that early recognition requires high clinical suspicion, meticulous documentation of symptom temporal patterns, and a low threshold for therapeutic trials when diagnostic uncertainty persists. Early recognition is critical to prevent complications from untreated autonomic storms. Although alternative diagnoses such as sepsis, seizures, and metabolic derangements were systematically excluded at the time of recurrent episodes, it is important to note that mimics and PSH can occasionally coexist in critically-ill patients. This highlights the need for ongoing reassessment and a high index of suspicion for other pathology even after PSH is diagnosed.

Therapeutic strategies

While no consensus guidelines exist for PSH, multimodal therapy is widely advocated. First-line interventions typically target cortical-subcortical disinhibition with GABAergic agents (e.g., baclofen, benzodiazepines), followed by second-line options of dopaminergic modulators (e.g., bromocriptine) [14]. In our patient, PSH was recognized by day five of admission after recurrent paroxysms persisted despite appropriate antimicrobial therapy and exclusion of other causes. Bromocriptine was initiated on the same day, reflecting relatively early therapeutic intervention once diagnostic confidence was achieved.

Supportive measures - including temperature control, hydration, and renal monitoring - are equally critical and were maintained throughout. Despite early intervention with bromocriptine, the patient developed rhabdomyolysis and acute kidney injury early in the course, before full therapeutic control was achieved. This suggests that delayed recognition and the cumulative burden of severe dystonic episodes contributed to muscle injury rather than treatment failure. On day 14, baclofen and clobazam were added in response to persistent dystonia despite bromocriptine, resulting in shorter and less severe episodes, reflecting a stepwise escalation strategy tailored to clinical response and indicating incremental benefit from combined GABAergic and dopaminergic modulation. Our patient’s improvement with this regimen aligns with evidence supporting dopamine modulation and GABAergic suppression of sympathetic overactivity [4].

A pragmatic clinical algorithm emerges from these observations: (i) early recognition of episodic autonomic features, (ii) exclusion of mimics, (iii) initiation of GABAergic and dopaminergic therapy, and (iv) monitoring for complications. Earlier escalation and proactive monitoring for rigidity and muscle injury may help prevent downstream complications such as rhabdomyolysis. This approach not only addresses the immediate autonomic storm but also mitigates downstream complications.

## Conclusions

PSH remains an underrecognized but clinically significant complication following brain injury, particularly in brainstem hemorrhages, where direct disruption of autonomic centers may lead to severe and refractory presentations. This case highlights the diagnostic challenges posed by symptom overlap with other common conditions and a lack of standardized criteria, often delaying appropriate treatment.

Our case illustrates the importance of high clinical suspicion, early recognition, and initiation of targeted therapy to prevent secondary insults. In our patient, stepwise therapy with bromocriptine, baclofen, and clobazam achieved partial control of sympathetic storms, demonstrating the potential benefit of combined dopaminergic and GABAergic approaches. While mortality in this case was ultimately due to hospital‑acquired pneumonia rather than PSH itself, delayed recognition likely contributed to secondary complications such as rhabdomyolysis and acute kidney injury.

Currently, management relies on empirical approaches with the combination of GABAergic agents and dopamine modulators for spinal hyperexcitability and diencephalic dysfunction. However, outcomes remain suboptimal, highlighting the need for evidence-based treatment protocols. Further studies are required to define reliable diagnostic tools, better characterize brainstem circuitry, evaluate therapeutic efficacy, and establish prognostic indicators to prevent complications and improve outcomes. Until then, vigilance, early intervention, and supportive care remain cornerstones of the management of PSH.
